# Analysis of the microecological mechanism of diabetic kidney disease based on the theory of “gut–kidney axis”: A systematic review

**DOI:** 10.1515/biol-2022-0909

**Published:** 2024-08-06

**Authors:** Lili Huang, Wenjing Wu, Xiaoqin Wang

**Affiliations:** Clinical College of Chinese Medicine, Hubei University of Chinese Medicine, Wuhan 430061, China; The First Clinical Medical School, Hubei University of Chinese Medicine, Wuhan, 430061, China; Department of Nephrology, Affiliated Hospital of Hubei University of Chinese Medicine, Hubei Provincial Hospital of Traditional Chinese Medicine, Wuhan, 430061, China; Hubei Key Laboratory of Theory and Application Research of Liver and Kidney in Traditional Chinese Medicine, Hubei Provincial Hospital of Traditional Chinese Medicine, Wuhan, 430061, China

**Keywords:** diabetic kidney disease, intestinal microbiota, metabolite, “gut–kidney axis”

## Abstract

Diabetic kidney disease (DKD) is one of the main microvascular complications of diabetes mellitus, as well as the leading cause of end-stage renal disease. Intestinal microbiota has emerged as a crucial regulator of its occurrence and development. Dysbiosis of the intestinal microbiota can disrupt the intestinal mucosal barrier, abnormal immunological response, reduction in short-chain fatty acid metabolites, and elevation of uremic toxins, all closely related to the occurrence and development of DKD. However, the underlying mechanisms of how intestinal microbiota and its metabolites influence the onset and progression of DKD has not been fully elucidated. In the current review, we will try to summarize the microecological mechanism of DKD by focusing on three aspects: the intestinal microbiota and its associated metabolites, and the “gut–kidney axis,” and try to summarize therapies targeted at managing the intestinal microbiota, expecting to provide theoretical basis for the subsequent study of the relationship between intestinal homeostasis and DKD, and will open an emerging perspective and orientation for DKD treatment.

## 1 Introduction

Diabetic kidney disease (DKD) is one of the most prevalent and severe complications of diabetes mellitus (DM) and is the leading cause of end-stage renal disease (ESRD). China is a major country with the largest number of diabetes patients in the world. According to the latest data from the International Diabetes Federation, approximately 783 million people worldwide will live with DKD by 2045, among which more than 147 million people will be from China. The disease incidence rate, mortality rate, and healthcare expenditure all show an increasing trend year by year. Clinical routine therapies include strict control of blood glucose and blood pressure, lipid-lowering therapy, and reducing urinary protein, which has a protective effect on the kidney but cannot prevent the progression of DKD to ESRD. Therefore, it is urgent for scholars to find new therapeutic targets for the pathogenesis of DKD and realize early detection and prevention.

The intestinal microbiota is symbiotic with the host and participates in various physiological activities. It is vital in fermenting food, resisting pathogens, maintaining metabolic homeostasis, and immune defense. The alterations in species, compositional, location, and functional alterations of beneficial and harmful bacteria, termed dysbiosis, may contribute to many metabolic ailments, including obesity and DM [1]. With the rapid advancements of sequencing technology and bioinformatics analytical techniques, Meijers and Evenepoel [2] proposed the concept of “gut–kidney axis,” stressing the bidirectional crosstalk between the kidneys and intestinal microbiota. Taking DKD as an example, the disorder of intestinal microbiota can induce the abnormal accumulation of uremic toxins such as indole sulfate, *p*-methyl sulfate, and trimethylamine oxide (TMAO), which can trigger a systemic inflammatory response, resulting in the deterioration of renal function. On the other hand, the decline in renal function will lead to numerous metabolites, such as uric acid and oxalate, which the kidney cannot entirely excrete, and as a result, they will accumulate, ultimately aggravating the disorder of intestinal microbiota via entering the intestinal lumen thereby contributing to disease progression [3]. Therefore, regulating intestinal microbiota and reversing dysbiosis to restore intestinal homeostasis is expected to be an appealing target for adjuvant therapy in DKD. Nevertheless, the mechanism of the dysbiosis of intestinal microbiota and its relationship to the progression of DKD are yet to be fully elucidated. The present study was conducted to take the “gut–kidney axis” as the starting point to explore the microecological mechanism of DKD to provide a scientific basis for clarifying its etiology and targeted therapy for microbiota dysbiosis.

## 2 Role of intestinal microbiota and its metabolites in the progression of DKD

### 2.1 Intestinal dysbiosis in DKD

As discussed earlier, DKD patients showed dysbiosis and a decrease in intestinal microbiota richness and diversity compared with healthy subjects, which usually manifests in an increase in the proportion of multiple pathogenic bacteria, a reduction in probiotics, and an increase in the number of Firmicutes/Bacteroidetes (F/B) ratio [4]. Firmicutes were reported to be negatively correlated with the degree of kidney damage in DKD [5], consequently, a decline in the Firmicutes may be a common feature. In terms of the genus level, patients with DKD usually displayed a decrease in the relative abundance of *Bifidobacterium*, *Lactobacillus*, and *Prevotella* and an increase in various opportunistic pathogens such as *Enterococcus faecalis* and *Desulfovibrio desulfuricans*. A metagenome-wide association study analysis revealed that the *Roseburia* in DKD patients was significantly decreased, while *Bacteroides stercoris* was enriched, and lack of some butyrate-producing bacteria (*Clostridium*, *Eubacterium* and *Roseburia intestinalis*) and potential probiotics (*Lachnospira* and *Intestinibacter*) [6]. According to the experimental results of Zeng et al. [7], *Prevotella* had the highest abundance in healthy subjects, while *Bacteroidetes* was the dominant bacterium in T2DM and DKD. As severe insulin resistance was related to a decreased relative abundance of *Prevotella* and an increase in *Bacteroides*, in this sense, the transformation of the dominant bacterium may play an essential role in the progression of DKD. It is worth mentioning that although the difference in intestinal microbiota dominantly came from the disease, the stage, gender, and age had some impact. A study of Chinese DKD patients revealed that the *Acidaminococcus*, *Fusobacterium*, *Prevotella_7*, *Sutterella*, and *Tyzzerella_3* showed a significant difference between the male and female DKD patients [8], and when patients were in DKD stage-3 or 4, the abundance of Gram-negative bacteria is enriched [9]. Since hypoglycemic, hypotensive, and hypolipidemic medicines and sample size, complicated primary diseases, and lifestyle habits such as male patients’ drinking and smoking could be important factors that modify the intestinal microbiome profile. Further systematic clinical studies are needed to explore these factors’ effects on the intestinal microflora (Table 1).

**Table 1:** Changes in intestinal microbiota composition and abundance in DKD

Subject	Changes in the composition and abundance		Functional effects and/or relevance	Reference
	Phylum level	Genus level		
Mice with DKD	Actinobacteria↑	*Prevotella*↓, *Bifidobacterium*↓, *Rikenella*↓, *Ruminococcus*↓, *Bacteroides*↓	Related to short-chain fatty acids (SCFAs) production	[17]
Patients with DKD	Actinobacteria↑	*Actinobacteria*↑, *Veillonella*↑, *Bifidobacterium*↑, *Clostridia*↓	Associated with SCFAs production and inflammation	[8]
Rats with DKD	Bacteroidetes↓	*Bacteroides*↓, *Lactobacillus*↓, *Escherichia*-*Shigella*↑, *Ruminococcus*_1↑	Involved in polysaccharide degradation and bile acid metabolism	[18]
Mice with DKD	Firmicutes↓	*Anaerosporobacter*↑, *Allobaculum*↑	Affects intestinal permeability and causes renal fibrosis	[19]
Rats with DKD	Firmicutes↓	*Turicibacter*↑, *Bifidobacterium*↑, *Desulfovibrio*↑, *Clostridium*↓, *Lactobacillus*↓	Leads to lipid and glucose metabolism disorders	[20]
	Actinobacteria↑			
	Proteobacteria↑			
Patients with ESRD	Actinobacteria↑	*Brachybacterium*↑, *Catenibacterium*↑	Damage intestinal barrier function and structure, leading to kidney inflammation	[21]
	Firmicutes↑			
	Proteobacteria↑			
Patients with ESRD	Proteobacteria↑	*Streptococcus*↑, *Prevotella*↓, *Roseburia*↓, *Faecalibacterium*↓, *Clostridium*↓, *Coprococcus*↓	Results in reduced butyrate production and thus increased uremic toxin production in ESRD patients	[22]
	Verrucomicrobia↑			
	Actinobacteria↓			
T2DM patients with DKD	Bacteroidetes↑	*Roseburia intestinalis*↓, *Bacteroides stercoris*↑, *Clostridium*↓, *Eubacterium*↓, *Roseburia*↓, *Lachnospira*↓, *testinibacter*↓	Participates in lipid metabolism and glucose metabolism in DKD patients, renal function is affected and the progression of DKD is aggravated	[6]
	Firmicutes↓			
	Proteobacteria↑			
Patients with DKD	Firmicutes↓	*Blautia*↓, *Lachnospira*↓, *Rosetella*↓, *Veillonella*↓, *Bifidobacterium*↑	Affects intestinal immune function and intestinal mucosal barrier	[23]
	Bacteroidetes↓			
	Actinobacteria↑			
Patients with DKD	Proteobacteria↑	*Escherichia-shigella*↑, *Proteobacteria*↑, *Prevotella*_9↓, *Prevotella*↓	Contribute to the physiological and pathological diagnosis of DKD	[24]
	Firmicutes↓			
Rats with DKD	Firmicutes↓	*Romboutsia*↓, *Turicibacter*↓, *Lactobacillus*↓, *Escherichia-Shigella*↑, *Ruminococcus*↑	Causes increased enterotoxin, intestinal damage, and insulin resistance	[25]
	Proteobacteria↑			
	Bacteroidetes↑			
Patients with DKD	Bacteroidetes↓	*Candidatus_Saccharimonas*↑, *Treponema*↑, *Desulfovibrio*↑, *Lactobacillus*↓, *Anaerovibrio*↓, *Bacteroides*↓	Triggers oxidative stress and inflammation in the kidneys	[26]
	Firmicutes↑			
Rats with DKD	Actinobacteria↑	*Turicibacter*↑, *Coprobacillus*↑, *Prevotella*↓, *Clostridium*↓, *Ruminococcus*↓, *Oscillospira*↓	Aggravated kidney injury	[27]
	Firmicutes↓			
	Proteobacteria↓			

### 2.2 Effects of intestinal microbiota-derived metabolites on the progression of DKD

Intestinal microbiota can directly participate in the metabolism of proteins, free amino acids, and carbohydrates and convert exogenous dietary substrates and endogenous host compounds into many small-molecule metabolites to achieve host-microbiota crosstalk and be correlated with kidney function [10]. The metabolites produced by the intestinal microbiota may also have pathogenic or beneficial effects on the host. Benefiting metabolites such as SCFAs, bile acids (BAs), and tryptophan (Trp) are critical mediators in the realization of host-microbiota crosstalk. When DKD progresses from the middle to advanced, renal filtration is impaired, and amino acid transit time in the colon is prolonged, increasing concentrations. This process induces an upstream expansion of protein-hydrolyzing species and increases the subsequent metabolism of aromatic amino acids, which become precursors of uremic toxins [11]. Some of these neurotoxins are of food origin and are synthesized by the intestinal microbiota, also known as “enterotoxins,” mainly indoxyl-sulfate (IS), *p*-cresyl sulfate (PCS), and TMAO. Microbiota-derived uremic toxins and their precursors are associated with variations in the makeup of the intestinal microbiota, which play an essential role in the pro-inflammatory response and the progression of DKD (Table 2).

**Table 2:** Effects of intestinal microbiota related metabolites on DKD

Metabolites	Source	Pathological/physiological mechanism
SCFAs	*Bififidobacterium*, *Faecalibacterium*, *Prausnitzii*, *Roseburia* sp. and^*Clostridium leptum*^[28]	Reduce the pro-inflammatory factors IL-1, IL-6, TNF-a, and block the activation of NF-kB pathway [29]
		Inhibition of histone deacetylase HDAC-mediated cellular NADPH oxidase Type 2/reactive oxygen species (ROS) signaling pathway reduces ROS production [30]
		Promote glucagon-like peptide-1 (GLP1) and recombinant peptide YY secretion, improve insulin sensitivity, inhibit gastric emptying [31]
		Protects the structural integrity of podocytes and reduces urine protein production [8]
BAs	*Lactobacillus*, *Bacteroides* and *Roseburia* [32]	Activation of Takeda G protein-coupled receptor 5 (TGR5) inhibits nuclear factor κB (NF-kB) signaling pathway and reduces inflammation and fibrosis in DKD [33]
		Improved insulin sensitivity via farnesoid X receptor (FXR) mediated signaling pathway [34]
		By activating the expression of BA membrane receptor 5 (TGR5) on the surface of islet B cells, as well as promotes insulin secretion and participates in the regulation of glucose metabolism [35]
Trp	*Escherichia coli*, *Lactobacilli*, *Ruminococcus* and *Clostridium sporogenes* [36,37]	Induces the release of GLP-1 to suppress appetite, promote insulin secretion, and slow gastric emptying [38]
		Maintaining intestinal mucosal homeostasis by regulating interleukin (IL)-22 release through indole-3-aldehyde (IAld)-mediated aryl hydrocarbon receptor (AHR) activation [38]
TMAO	Enterococcus, Betaproteobacteria, Sporosarcina, Klebsiella pneumoniae, Providencia and Shigella [39]	Activation of the mitogen-activated protein kinase/NF-κB signaling pathway produces inflammatory cytokines [40]
		Down-regulating the mRNA expression of phosphatidylinositol 3-kinase (PI3K) and Akt leads to the increase in blood glucose [40]
		Induction of conversion factor-β promoted myofibroblast transformation [41]
IS	*Escherichia coli*, *Proteus vulgaris*, *Paracolobactrum coliforme*, *Bacteroides* spp, *Achromobacter liquefaciens* [42,43]	Activating NF-kappa B26 and inhibiting the expression of anti-aging gene klotho accelerated cell aging and renal tubule interstitium fibrosis [44]
		Chronic activation of AHRs by high levels of IS leads to progressive damage to podocytes and glomeruli [45]
		Restriction of endothelial cell proliferation leads to renal endothelial dysfunction and nitric oxide production, inducing oxidative stress and senescence of endothelial cells [46]
PCS	Bacteroides, Bacteroidaceae, Bifidobacteriaceae, Clostridiaceae, Enterococcaceae, Eubacteriaceae [47,48]	Up-regulation of nuclear factor κB triggers inflammation in the body [49]
		Upregulated mRNA levels and TGF-β1 secretion lead to tubular cell damage, tubular interstitial inflammation and fibrosis [49]
		Increasing the expression of angiotensinogen (AGT) and angiotensin receptor AT1R, while decreasing the expression of angiotensin receptor AT2R aggravated oxidative stress [50]
PS	Clostridiaceae, Enterococcaceae, Staphylococcaceae, Bacteroidaceae, Bifidobacteriaceae, and Enterobacteriaceae [51,52]	Leads to podocyte damage and directly induces proteinuria [53]
		Reduces the level of low glutathione, causing oxidative stress and damage to cells [53]
H2S	*Desulfovibrio*, *Desulfomonas*, Desulfobulbus and Desulfotomaculum [54]	Improve and regulate renin, reverse the synergistic effect of angiotensin type II (AngⅡ) and hyperglycemia, and improve renal hemodynamics [55]
		Inhibition of mitochondrial cytochrome c oxidase activity, prevent inflammation and renal fibrosis [56]

#### 2.2.1 SCFAs

SCFAs are significant metabolites of intestinal microbiota, produced by anaerobic bacteria that can ferment undigested carbohydrates, such as *Bacteroides*, *Bifidobacterium*, *Clostridium*, and *Streptococcus.* Among them, acetate, propionate, and butyrate are the most abundant. SCFAs demonstrated to modulate immunoreaction and inflammatory responses by inhibiting the activity of histone deacetylase (HDAC) and binding to G-protein-coupled receptors such as GPR43, GPR41, and GPR109A in intestinal epithelial cells to protect against the development of DKD [12], are established to be crucial molecules in the regulation of host-microbiota. Due to the restriction of high-fiber diets and fruit intake, DM patients are usually accompanied by a reduction in SCFAs-producing bacteria as well as low serum and fecal SCFA levels, which can diminish the ability to activate receptors and may be correlated with the development of DKD [13]. Butyrate was reported to down-regulate angiotensin II-induced expression of renin and its receptor, thus ameliorating renal damage induced by angiotensin II [14]. Interestingly, the study of Martin et al. [15] found that in obese or DKD mice, strict dietary restrictions and drug treatment had little effect on fasting blood glucose and body weight. However, DKD or obesity was significantly alleviated after prolonged administration with acetate or butyrate. Another experimental data [16] also established that butyrate could effectively inhibit the inflammatory response to protect the kidney in DKD mice by activating the AMP-activated protein kinase (AMPK) signaling pathway and promoting glucagon-like peptide-1 receptor (GLP-1R). Consequently, oral supplementation of SCFAs (especially butyrate) may act as an effective strategy for treating DKD. However, due to the lack of uniform standards for the dose and concentration of SCFAs for oral and fewer known risks of long-term supplementation, most of the above data are derived from animal experiments, requiring more studies to demonstrate whether these experimental results can be reproduced in humans.

#### 2.2.2 BAs

BAs are synthesized from cholesterol in hepatocytes and then metabolized by the intestinal microbiota via the processes of uncoupling, dehydrogenation, dihydroxylation, oxidative reactions, and epimeric isomerization, which converts primary BAs to secondary BAs [57]. Most of them are reabsorbed by the liver, with a small portion entering the circulation. BAs are ligands for TGR5 and FXR, both of which regulate DKD and are mainly reflected in glucolipid metabolism and energy metabolism [58]. In the early DKD, BAs combine with TGR5 to improve insulin sensitivity via GLP-1, as well as activate glucosyl-induced phosphorylation through FXR-mediated signaling pathways, increase the ratio of ATP/ADP, and increase cellular oxygen consumption, while intracellular calcium ion flow induces active insulin output to the outside of the cell, resulting in maintaining insulin homeostasis in the body [59]. In the middle and late stages, BAs can activate FXR and TGR5 to inhibit the inflammatory response and exert renoprotective effects. Among them, activation of TGR5 reduces macrophage inflammatory response, inhibiting the expression of pro-inflammatory factors in monocytes. In *in vivo* experiment, it was illustrated that [60] the expression of several pro-inflammatory factors such as tumor necrosis factor-α (TNF-α), IL-6, and monocyte chemotactic factor (MCP-1) were down-regulated in kidneys after intraperitoneal injection of the FXR agonist GW4064 in db/db mice. These studies suggest that FXR has the potential to serve as one of the effective therapeutic targets for DKD, and a clear causal relationship between FXR activation and DKD is necessary to be established in the future.

#### 2.2.3 Tryptophan

Trp, an essential aromatic amino acid that originates from dietary proteins, is the only amino acid with an indole structure. Although most of the proteins are digested and absorbed mainly in the small intestine, there are still about (6–18 g/day) of proteins and amino acids decomposed by the microbiota after reaching the colon [61]. When protein intake is excessive, colonic carbohydrates are depleted, accompanied by increasing pH and prolonged emptying time, all of which provoke the catabolism of proteins by the intestinal microbiota shifting from sugar hydrolysis to protein fermentation. The compounds produced by Trp metabolism, including tryptamine, methyl indole, indole acetic acid (IAA), indole acrylic acid (IA), indole aldehyde (IAld), and indolyl lactic acid, are ligands for the AHR, and could induce AHR conformational changes, thus affecting innate and adaptive immune responses. IAld, e.g., contributes to maintaining intestinal mucosal homeostasis by inducing an increase in interleukin-22 (IL-22) production through AHR activation [62]. Tryptamine induces the release of 5-hydroxytryptamine from enterochromaffin cells, and indole induces the release of GLP-1, which suppresses appetite, promotes insulin secretion, and slows gastric emptying [38]. Besides intestinal metabolism, the kidney is vital in amino acid synthesis, degradation, conversion, and tubular reabsorption. Chou et al. [63], analyzing plasma metabolites in patients with different stages of DKD, found that Trp was significantly correlated with a rapid decrease in glomerular filtration rate and was the most valuable predictor of DKD when serum Trp concentration was below 44.20 μM (sensitivity 55.6% and specificity 87%), which provides direct evidence for Trp to assess the prognosis of DKD. The accuracy of glomerular filtration rate in assessing end-stage renal dysfunction has been questioned because renal hyperfiltration triggers abnormal elevation of glomerular filtration rate; it gives the promise that lower levels of Trp (below 44.2 μM) can be a reliable alternative marker for predicting the prognosis of DKD.

#### 2.2.4 TMAO

TMAO is an indirect metabolite of intestinal microbiota; the intestinal microbiota first ferments diet-derived choline, phosphatidylcholine (lecithin), and l-carnitine to produce trimethylamine (TMA), which is then oxidized into TMAO by enzyme flavin-containing monooxygenase 3 in the liver. TMAO has emerged as an independent risk factor for kidney injury and is used in clinical studies to predict mortality and prognosis of cardiovascular events in type 1 diabetes. A more recent study published in “Nature Communications” [41] analyzed circulating TMAO concentrations and various body phenotypes found that intake of foods rich in TMAO precursors, such as red meat, eggs, and milk, did not lead to an increase in circulating levels of TMAO, which are primarily determined by renal clearance. Recent studies generally agree that TMAO can directly cause renal dysfunction by causing kidney fibrosis and promoting inflammation and oxidative stress, all crucial elements in the pathogenesis of DKD. In animal models, TMAO can promote DKD by aggravating renal fibrosis via inducing transforming growth factor-β (TGF-β), activating the NOD-like receptors family pyrin domain containing-3 (NLRP3) inflammasome and resulting in the release of IL-1β and IL-18 to accelerate renal inflammation [40]. Supplementation with TMA inhibitors was found to reduce plasma TMAO levels significantly and prevent renal functional impairment and fibrosis [64]. Based on the above studies, it is known that high TMAO levels can exacerbate DKD, and TMAO inhibitors may have therapeutic potential to ameliorate renal injury in DKD.

#### 2.2.5 IS

IS originates from the metabolism of tryptophan by intestinal microbiota, where dietary tryptophan is metabolized by *E. coli* and *Bacteroides*, among others, in the intestinal tract to produce indole, which passes from the portal vein to the liver, where it undergoes hydroxylation and sulfation to produce IS [65] ultimately. High levels of IS can lead to enhanced oxidative stress and increased intestinal epithelial permeability, accelerated cellular senescence through activation of NF-kappa B26, and mediates tubulointerstitial fibrosis through inhibition of the expression of the anti-aging gene klotho [44]. In addition, IS induces altered podocyte morphology, increased expression of pro-inflammatory cytokines and chemokines, and glomerular injury, ultimately leading to progressive decline in renal function. A study comparing IS concentrations in the serum of type 2 diabetic patients with or without renal impairment [66] found that the levels of serum creatinine, proteinuria, and IS in patients with renal injury were significantly higher than those in patients without nephropathy and the glomerular filtration rate was significantly lower. Another study analyzing IS levels in the serum of patients with different CKD stages found [67] that serum IS levels tended to increase gradually with the progression, showed a strong negative correlation with glomerular filtration rate, and reached a peak in patients on maintenance dialysis. Notably, the IS can be clear by residual renal function to a certain extent in patients on abdominal dialysis, suggesting that protection of the residual renal function contributes to the clearance of protein toxins.

#### 2.2.6 PCS

PCS is a product of fermentation of tyrosine and phenylalanine by intestinal microbiota in the colon. When urea levels are elevated, the urease-producing species such as Clostridiaceae, Verrucaceae, and Enterobacteriaceae are enriched in the intestine [68], which participate in the formation of *p*-cresol and indole, the latter of which is a precursor of IS, through the expression of urease. Serum PCS levels are negatively correlated with renal function; on the one hand, PCS triggers the body’s inflammatory response through the upregulation of NF-κB, as well as induces renal tubular cell injury, tubulointerstitial inflammation, and fibrosis through the upregulation of mRNA levels and TGF-β1 secretion, which further results in the decline of renal function [49]. At the same time, when renal function was impaired, PCS significantly decreased peripheral B lymphocytes while increasing the IL-10, which in turn inhibits macrophage activity through negative feedback, leading to failure of adaptive immune response in DKD [69]. The above results suggest that PCS indirectly leads to host immune dysfunction mainly by disrupting the intestinal barrier function, both of which contribute to maintaining a pro-inflammatory condition. Since both PCS and IS are protein-bound toxins, which are bound to proteins *in vivo* and have a large relative molecular mass, making it difficult to remove them adequately by dialysis, leading to their deposition and increased toxicity in patients with ESKD, the intestinal microbiota may be a future target for decreasing the levels of the precursors of these toxins and their toxicity, as well as slowing down the onset of the development of DKD in its early stages.

## 3 Connotation of the “gut–kidney axis” theory in DKD

**Figure 1:**
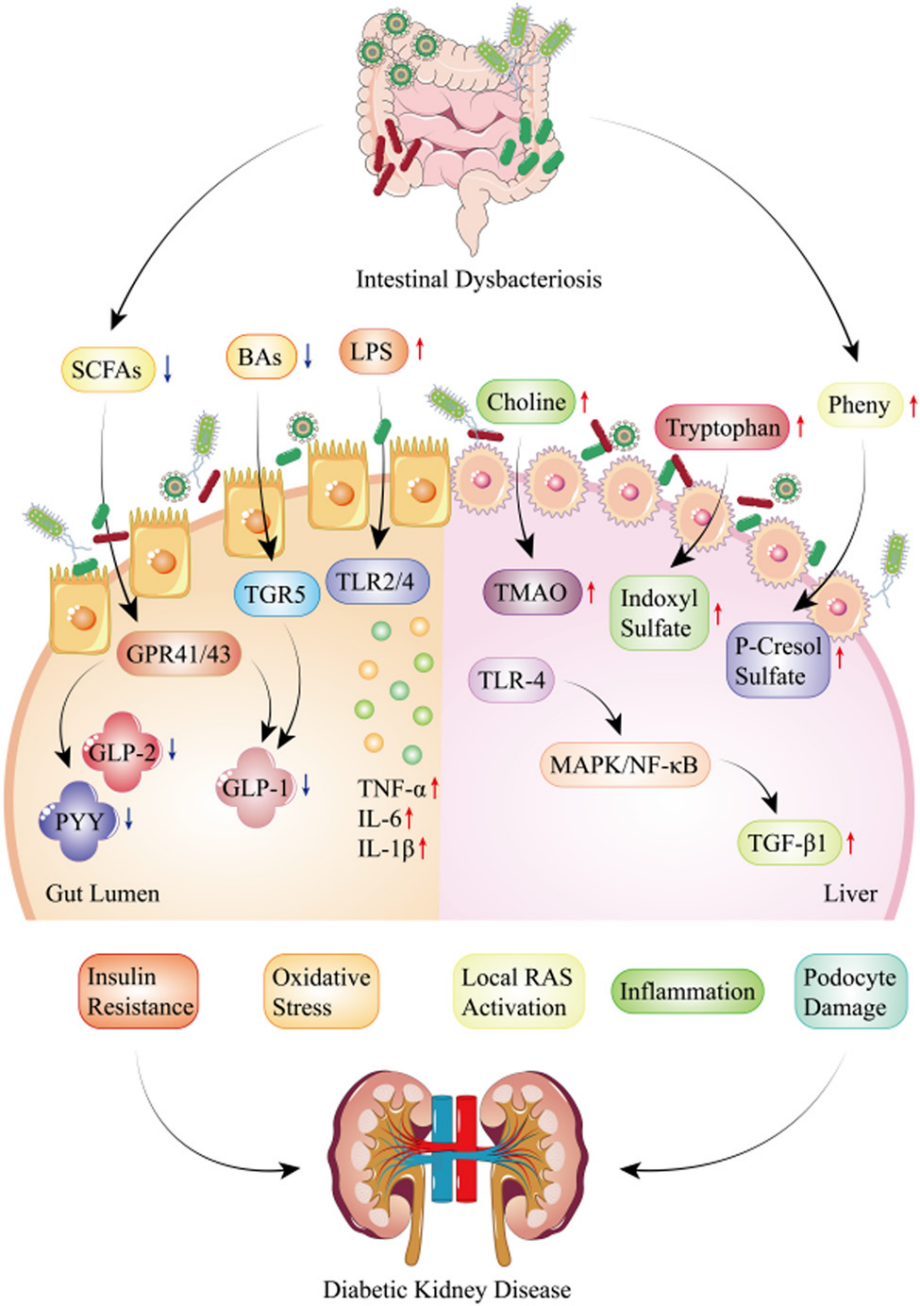
The “Gut–Kidney Axis” in DKD: In the case of DKD, the decline in renal function increases circulating uremic toxins and affects the composition and function of intestinal microbiota. A decrease in beneficial bacteria leads to reduced production of SCFA, BAs, and Tryptophan. At the same time, harmful bacteria increased, and their toxic metabolites, such as IS, LPS, PCS, and TMAO, were released into the circulation through the damaged intestinal mucosal barrier, activating a series of immune-inflammatory reactions, leading to insulin resistance and kidney function damage, ultimately deteriorating DKD.

The “gut–kidney axis” theory emphasizes the interaction between the gastrointestinal tract and the kidney, which can be subdivided into metabolism-dependent and immune pathways, with the metabolism-dependent pathway mediated by intestinal microbiota and its subsequent metabolites. In the case of DKD, reduced renal filtration rate causes the deposition of many nitrogen-containing products, such as α-amino nitrogen, in the circulation, which were metabolized by intestinal microbiota into uremic toxins. Typically, these toxins are excreted by organic anion transporters (OATs) in renal tubule epithelial cells. However, once uremic toxins like indoxyl sulfate, *p*-cresol, and *p*-cresol sulfate enter renal tubular cells through OATs, they induce oxidative stress and inflammation both locally in the tubules and glomeruli and further destroy intestinal tight junction proteins such as claudin 1, occludin, and zonula occludens 1 (ZO-1) with increasing concentration. When the intestinal barrier is broken down, harmful bacteria and their toxins enter the circulation, causing kidney and even systemic inflammation [70], ultimately accelerating kidney function deterioration. Thus, according to the “gut–kidney axis,” intestinal microbiota homeostasis, intestinal mucosal barrier function, and immune-mediated inflammation are three essential intermediaries of crosstalk between gut health and kidney function (Figure 1).

### 3.1 Impaired intestinal barrier function

As the first line of defense between the intestinal environment and the outside, intestinal mucosal barrier is the most important link of intestinal defense mechanism, and its integrity is related to tight junction proteins. The disturbance of intestinal microbiota leads to the contraction and entosis of critical tight junction proteins such as occlusion and ZO-1 [71], resulting in increased intestinal permeability and translocation of bacterial metabolites across the intestinal barrier. During this time, large amounts of accumulated urea metabolites like NH₃ in the gut are absorbed into the bloodstream and then resynthesize urea in the liver, triggering an inflammatory response of the intestinal immune system [72]. Additionally, intestinal epithelial also expresses pro-inflammatory cytokines, promotes responses to Th1 and Th17 via dendritic cells and macrophages, and produces higher levels of symbion-specific IgG via B cells. At the same time, cresol, indolyl molecules, and other toxins are transferred into the blood and accelerate the deterioration of DKD by triggering tissue inflammation and the production of reactive oxygen species [73]. It is clear that the accumulation of uremic toxins is considered to be a crucial factor leading to impaired intestinal barrier function and triggering the intestinal immune system.

### 3.2 Immune inflammatory response

A chronic low-grade inflammatory state due to immune dysregulation and sustained production of pro-inflammatory cytokines is a prominent feature of DKD [74]. During dysbiosis with bacterial translocation and increased intestinal permeability, metabolic endotoxin/LPS dysregulation continuously leaks into the portal vein via the intestinal mucosal barrier. LPS is a surface antigen of Gram-negative bacteria that induces downstream signaling cascades by binding to TLR2/4. For one thing, binding of LPS to TLRs activates the renal medullary differentiation MyD88/NF-κB signaling pathway [75], which is considered to be the master switch controlling inflammation when in a high-glycemic environment, NF-κB activity is elevated, with an enhanced expression of pro-inflammatory factors such as TNF-α, IL-6, and cell adhesion proteins, increased levels of inflammatory cytokines in both the bloodstream and kidney, and activation of the innate immune system. Further, the activation of TLRs also promotes the secretion of transforming growth factor-β (TGF-β), which mediates the proliferation of renal mesangial cells, glomerulosclerosis, and renal interstitial fibrosis [76]. It has been demonstrated that in TLR2 receptor-deficient mice, the MyD88 signaling pathway was attenuated, and TLR4 receptor expression could be blocked by reduced macrophage aggregation and pro-inflammatory cytokines [77]. There is also evidence that, in the experimental DKD mouse model lacking the TLR4 receptor, renal NF-κB activation was reduced, which could reverse renal dysfunction to achieve renal protection [78]. In summary, LPS disrupts the intestinal mucosal barrier and binds to renal TLRs to activate the MyD88/NF-κB signaling pathway, which was suggested as the critical mechanism by which intestinal microbiota mediate the inflammatory response to DKD.

## 4 Regulation of intestinal microbiota in the treatment of DKD

Increasingly emphasized correlation between DKD and intestinal dysbiosis makes remodeling of the intestinal microbiota to regulate the “gut–kidney axis” a promising therapeutic candidate for preventing and managing DKD and its metabolic complications. Exogenous intervention measures such as diet adjustment, fecal flora transplantation, genetically engineered bacteria, and endogenous supplement microorganisms such as oral probiotics, prebiotics, and epigenetics have been proposed successively. However, there still remains a significant challenge for translating microbiome findings into biologically relevant ones. For example, the survival rate of oral probiotics in the gastrointestinal tract is only 20–40% due to gastric and BAs [79], which makes it difficult for them to colonize the intestinal mucosa, and little is known about their biological host response. To address this question, Zmora et al. [80] proposed a therapy of “personalized microbiome”; thus, specific probiotics with known metabolic functions could be considered in the future. Apart from endogenous supplementation of microbial agents, fecal microbial transplantation, as an exogenous intervention, can realize the reconstruction of the intestinal microbial by transplanting probiotics from the intestinal tracts of healthy individuals to patients with dysbiosis. However, its technology has not yet been fully applied in the clinic, so more clinical data are needed to validate its effectiveness. Intriguingly, Traditional Chinese medicine (TCM) has provided strong evidence for the unique advantage of the “gut–kidney axis” in treating DKD, such as beneficially reshaped intestinal microbiota ecology and improved dysbiosis, promoting the expansion of SCFA-producing bacteria, which increased SCFA (especially butyrate) concentrations and reduced the production of uremic toxins in renal injury in patients with DKD [81,82]. Thereby researchers have increasingly turned their attention on treatment for DKD in the field of TCM. Considering these findings, Shen et al. [83] combined metabolomics and reverse transcriptomics and found that Yishen Huashi Granules could effectively treat DKD by regulating the levels of serum metabolites (phosphatidylethanolamine) and the expression of mRNAs (phospho1) in the kidneys, furthermore, improve the disorders of glucose–lipid metabolism and renal injury caused by DKD. Similarly, Du et al. [84] found that Shenqi Dihuang Decoction could enrich beneficial bacteria such as *Bifidobacteria* and *Bacteroides* to improve renal fibrosis and protect renal function. Nonetheless, due to the Chinese herbal compound’s multiple components, targets, and pathways characteristics, the specific molecular mechanism of its regulation of the “gut–kidney axis” in treating DKD still needs to be further elucidated.

## 5 Conclusion

Nowadays, the study of the gut–kidney crosstalk in DKD is receiving increasing concern. In this view, we combined microbiome and metabolomics, took the “gut–kidney axis” as the entry point, and reviewed the multiple pathways and targets of DKD regulation by intestinal microbes through its mediated inflammatory, immune, and metabolic pathways. It will open an emerging perspective and orientation to reveal the microecological mechanism and TCM therapeutic strategy of DKD. However, most current studies only prove the interaction between intestinal microbes and their metabolites with DKD, and there is a lack of causal studies. To this end, it is necessary to fully understand the pathophysiology of DKD from a large-scale prospective cohort (especially multi-omics data from the same patient cohort). To confirm the changes in the bacteria and its metabolites during the development of DKD, and to continue to explore specific strains of bacteria and microbial metabolites that can be used for the diagnosis and treatment of DKD, and based on this to investigate further the molecular mechanisms by which interactions between drugs, bacteria, and metabolites are realized through *in vitro* experiments.
